# Adaptive Associations between Social Cognition and Emotion Regulation are Absent in Schizophrenia and Bipolar Disorder

**DOI:** 10.3389/fpsyg.2012.00607

**Published:** 2013-01-11

**Authors:** Jesseca E. Rowland, Meelah K. Hamilton, Nicholas Vella, Bianca J. Lino, Philip B. Mitchell, Melissa J. Green

**Affiliations:** ^1^School of Psychiatry, University of New South WalesSydney, NSW, Australia; ^2^Black Dog Institute, Prince of Wales HospitalRandwick, NSW, Australia; ^3^Schizophrenia Research InstituteDarlinghurst, NSW, Australia

**Keywords:** social cognition, emotion, cognitive emotion regulation, schizophrenia, bipolar disorder

## Abstract

Schizophrenia (SZ) and bipolar disorder (BD) are associated with impairments in facial emotion perception and Theory of Mind (ToM). These social cognitive skills deficits may be related to a reduced capacity to effectively regulate one’s own emotions according to the social context. We therefore set out to examine the relationship between social cognitive abilities and the use of cognitive strategies for regulating negative emotion in SZ and BD. Participants were 56 SZ, 33 BD, and 58 healthy controls (HC) who completed the Ekman 60-faces test of facial emotion recognition; a sub-set of these participants also completed The Awareness of Social Inference Test (TASIT) and the Cognitive Emotion Regulation Questionnaire (CERQ). SZ participants demonstrated impairments in emotion perception on both the Ekman and the TASIT Emotion Evaluation tests relative to BD and HC. While both SZ and BD patients showed ToM deficits (i.e., perception of sarcasm and lie) compared to HC, SZ patients demonstrated significantly greater ToM impairment compared to BD. There were also distinct patterns of cognitive strategies used to regulate emotion in both patient groups: those with SZ were more likely to engage in catastrophizing and rumination, while BD subjects were more likely to blame themselves and were less likely to engage in positive reappraisal, relative to HC. In addition, those with SZ were more likely to blame others compared to BD. Associations between social cognition and affect regulation were revealed for HC only: TASIT performance was negatively associated with more frequent use of rumination, catastrophizing, and blaming others, such that more frequent use of maladaptive cognitive emotion regulation strategies was associated with poor social cognitive performance. These associations were not present in either patient group. However, both SZ and BD patients demonstrated poor ToM performance and aberrant use of emotion regulation strategies consistent with previous studies. SZ also showed basic emotion recognition deficits relative to BD and HC. That there were no associations between social cognition and the capacity to self-regulate negative emotion in SZ and BD (in the context of poor social cognition and maladaptive regulatory strategies) suggests that dysfunction in fronto-limbic brain networks may underpin both social cognitive deficits and the use of maladaptive cognitive strategies in these disorders, albeit by potentially different routes.

## Introduction

Despite their traditional conceptualization as separate diagnostic entities, there is growing recognition of overlapping pathology between schizophrenia (SZ) and bipolar disorder (BD), evident in shared clinical features (Murray et al., 2004), neurocognitive (Reichenberg et al., 2009; Bora et al., 2010), social cognitive deficits (Montag et al., 2010; Sparks et al., 2010), and genetic determinants (Lichtenstein et al., 2009). However, there remain obvious points of departure in the clinical pathology of SZ and BD, most notably in the overt expression of affect. That is, while BD is characterized by disturbance of mood reflected in manic and depressive states (Malhi et al., 2004a,b), overt manifestations of emotionality in SZ are often characterized by inappropriate or blunted affect, that is, lack of context-appropriate emotional expressivity (Gur et al., 2006).

Unique dysfunction in the brain networks required for the voluntary regulation of emotion in BD and SZ (Morris et al., 2012) suggest that the shared tendency to implement *maladaptive* regulatory styles (Rowland et al., in revision) may reflect distinct fronto-limbic brain network abnormalities. Specifically, Morris et al. found that patients with BD demonstrated increased but apparently ineffectual cortical activation during attempts to regulate negative emotion, while SZ patients generally failed to engage cortical regions during attempts to down-regulate negative emotion. In the context of established brain mechanisms responsible for the regulation of affect (Ochsner et al., 2004), it is possible that common cognitive deficits in SZ and BD (predominantly reflecting prefrontal cortical brain dysfunction) could impede the capacity for effective self-regulation of emotion in these disorders (Green et al., 2007). For instance, a review by Ochsner and Gross (2008) highlights a model of brain networks supporting cognitive emotion regulation where cognitive strategies vary in their reliance on prefrontal and cingulate systems for attention, response-selection, and mental-state attribution. These same deficits in executive function have been shown to influence mentalizing ability and facial affect processing in SZ and BD (Addington and Addington, 1998; Bora et al., 2005; Olley et al., 2005), such that there is potential for social cognitive deficits to also impact emotion regulation capacities in these clinical groups (Ochsner, 2008; Phillips et al., 2008). In this study we aimed to address this question in the context of known aberrations in both social cognition and emotion regulation in these disorders.

Social cognition is a domain of cognitive processing which involves all of the processes necessary for interacting with conspecifics (Adolphs, 2004), and encompasses two important skills: (1) the ability to accurately perceive emotional information from others (e.g., from facial expressions and vocal inflections); and (2) the ability to make higher order social inferences about the intentions and behavior of others, often referred to as Theory of Mind (ToM; Pinkham et al., 2003). Effective social cognitive processes are required to respond appropriately in social interactions (McDonald et al., 2003). Disrupted social cognition is frequently observed in SZ (Brune, 2005), with a considerable body of research demonstrating impairments in the perception of facial affect (Mandal et al., 1998; Edwards et al., 2002; Namiki et al., 2007; Kohler et al., 2010; Sparks et al., 2010) and emotional prosody (Leitman, 2005; Hoekert et al., 2007). There has been some evidence to suggest that SZ patients have more difficulty interpreting negative emotions (such as sadness and disgust) compared to positive emotions (such as happiness; Edwards et al., 2002). A bias to misinterpret “neutral” faces as “sad,” “happy,” or “disgusted” has also been demonstrated (Kohler et al., 2003). Furthermore, these impairments have been shown to persist during remission of psychotic symptoms (Kern et al., 2009). Similarly, studies of BD reveal that bipolar patients are also impaired on tasks of facial affect perception compared to healthy controls (HC), but do not perform as poorly as SZ patients (Addington and Addington, 1998). Indeed, meta-analyses of emotion perception in both patient groups have revealed a large deficit for SZ (Kohler et al., 2010) compared to a moderate deficit for BD (Kohler et al., 2011).

Interestingly, many studies suggest that mood-congruent biases in emotion perception may be operating in BD. For example, a “negative bias” has been found in BD during a depressed state, where there is a tendency to rate “neutral” faces as “sad” and “happy” faces as “neutral” (Gur et al., 1992; Gray et al., 2006). Comparable research during mania suggests a “positive bias” in which “neutral” facial expressions are interpreted as “happy” (Lennox et al., 2004). Furthermore, the persistence of social cognitive impairments into the euthymic phase of BD has also been reported (Yurgelun-Todd et al., 2000; Lembke and Ketter, 2002; Samame et al., 2012), although these findings are inconsistent (Malhi et al., 2007). In this study, the Ekman 60-faces task from the Facial Expressions of Emotion: Stimuli and Tests (FEEST; Young et al., 2002) was chosen as a well-validated facial affect processing task that requires participants to identify basic emotions from a series of faces presented in a still photographic format.

Static photographs, however, are markedly different to spontaneous dynamic displays of emotion that must be rapidly decoded in everyday social situations where emotions are displayed briefly and quickly. The Awareness of Social Inference Test (TASIT; McDonald et al., 2003) addresses this issue by using videotaped footage of trained actors in dynamic displays of emotion, as well as in conversational interactions with others, requiring the integration of multiple, changing cues (from face, prosody, gesture and context) to identify the emotions, beliefs, and intentions of target characters within the social context. The TASIT thus provides an ecologically valid measure of social cognition that assesses both simple (basic emotion perception) and complex (ToM skills) social cognition, with higher levels of difficulty requiring the integration of contextual cues and the perception of lies and sarcasm. Sarcasm is one example of a non-literal language device used in conversation that relies on ToM, since the true state of affairs is the opposite to that asserted in the literal utterance (Brown and Levinson, 1978; Haverkate, 1990). Recent studies using the TASIT demonstrate impairments of sarcasm perception in SZ (Leitman et al., 2006; Kern et al., 2009; Sparks et al., 2010), while studies of ToM deficits using more traditional picture-story tasks have repeatedly demonstrated ToM impairments in schizophrenic individuals (Brune, 2005; Harrington et al., 2005). Again, these deficits have been shown to persist when patients are in remission (Sprong et al., 2007). Mounting evidence also indicates that BD patients are impaired in their ability to “mentalize”: BD patients in an affective state (depressed or manic) have been found to perform worse on ToM tasks than euthymic BD patients who had comparable results to controls (Kerr et al., 2003). However, other studies have reported impaired ToM in euthymic BD as well (Bora et al., 2005; Montag et al., 2010).

The propensity for social cognitive deficits to impact emotion regulation is supported by current knowledge of the cognitive and neural mechanisms for emotion generation and regulation (Green and Malhi, 2006). Specifically, emotion regulation refers to a range of voluntary and involuntary processes used to modulate the occurrence, intensity, and duration of internal feeling states and physiological processes that occur in relationship to external events, in order to respond appropriately in accord with one’s goals (Gross, 1998; Eisenberg, 2000). Emotional responses are refined through a complex interaction between primitive limbic structures (engaged in the perception and generation of emotion) and cortical regions (engaged in inhibitory control of affective responses) to provide flexibility in response to changes in both internal and external environments (Green and Malhi, 2006). Common strategies to regulate subjective affect include attempts to cognitively control the type and extent of emotional response via techniques to reframe the meaning of the event (Ochsner and Gross, 2005); cognitive reappraisal is one such technique that involves reappraisal of emotional and social information to modulate one’s own emotional responses (Green and Malhi, 2006).

Cognitive reappraisal requires effective inhibition of the limbic system by prefrontal brain regions, specifically the orbitofrontal cortex (OFC), dorsolateral prefrontal cortex (DLPFC), lateral prefrontal cortex (LPFC), and medial prefrontal cortex (MPFC; Green and Malhi, 2006), such that known abnormalities in neurocognitive and social cognitive deficits in SZ and BD are therefore likely to impact mechanisms for emotion regulation.

For instance, working memory deficits in SZ and BD have also been associated with abnormal brain activity in the OFC and DLPFC (Park et al., 1999), and evidence has shown that social cognitive skills are partially accounted for by non-social neurocognitive capacities (Sergi et al., 2007), particularly those associated with prefrontal cortical function (Crowe et al., 1999). Furthermore, neuroimaging studies of social cognition in patients with mood disorders have revealed enhanced activation in limbic structures and attenuated activity within frontal regions associated with emotion regulation (Cusi et al., 2012). Indeed, we have recently demonstrated distinct aberrations in prefrontal-limbic brain networks in SZ and BD during attempts to down-regulate negative affect using cognitive reappraisal (Morris et al., 2012), In addition, emerging evidence suggests that “exhaustion” of these inhibitory regions can result in over-activation of the amygdala, alongside aberrant connectivity of PFC-amygdala networks (Wagner and Heatherton, 2012). These brain disturbances in networks required for efficient emotion regulation may explain why individuals with a history of psychosis demonstrate less frequent use of cognitive reappraisal relative to non-patient controls (Livingstone et al., 2009). Furthermore, with overt signs of dysregulated emotion in SZ most commonly seen in blunted affect, it is not surprising that attenuated affect in SZ has been associated with difficulties in the amplification (up-regulation) of positive emotional expression (Henry et al., 2007).

Direct comparison of SZ and BD with regard to cognitive processes used to regulate emotion has been recently undertaken in a large sample of outpatients assessed with the Cognitive Regulation of Emotion Questionnaire (CERQ; Rowland et al., in revision). This study revealed a pattern of similarly increased tendencies to employ rumination, self-blame, and catastrophizing evident among SZ and BD, alongside distinct use of other-blame in SZ (Rowland et al., in revision). The CERQ (Garnefski et al., 2001) provides an index of the extent to which particular cognitive strategies are employed to regulate negative emotion in response to threatening or stressful life events. Previous research using this scale has shown increased use of rumination, catastrophizing, and self-blame, coupled with decreased use of adaptive cognitive reframing strategies (for example, positive reappraisal), in association with depression and anxiety symptoms (Garnefski et al., 2001, 2002, 2005; Garnefski and Kraaij, 2006). A similar pattern of responses on the CERQ has also recently emerged for BD patients and their unaffected relatives (Green et al., 2011).

On the basis of likely inter-relationships among social cognitive ability and emotion regulatory capacities, we set out here to examine the relationship between both emotion perception and ToM disturbances and the use of cognitive strategies for regulating negative emotion in SZ and BD. The following hypotheses were tested on the basis of evidence reviewed above. First, we hypothesized that SZ and BD participants would both be impaired on the Ekman 60-faces task and TASIT emotion perception relative to controls, but with SZ patients showing greater impairment than those with BD. Second, it was hypothesized that both SZ and BD participants would be impaired on TASIT social inference tests relative to controls, with SZ participants again demonstrating greater impairments compared to BD participants. Third, we hypothesized that SZ and BD groups would be more likely to use maladaptive cognitive emotion regulation strategies (i.e., increased rumination, catastrophizing, and self-blame) and less likely to use adaptive positive reframing strategies (e.g., positive reappraisal) in comparison to controls. Lastly, we hypothesized that performance on the social cognition tasks would be associated with the use of particular CERQ strategies in the SZ and BD groups; specifically, that poorer performance on the Ekman task and TASIT would be associated with increased use of maladaptive emotion regulation strategies (rumination, self-blame, other-blame, and catastrophizing) and decreased use of adaptive reframing strategies (e.g., positive reappraisal).

## Materials and Methods

Study procedures were approved by the Human Research Ethics Committees of the University of New South Wales (HREC UNSW Protocol No. 07167) and the South Eastern Sydney Illawarra Area Health Service (SESIAHS Protocol No. 09/081). All participants provided written informed consent prior to participation.

### Participants

The sample comprised 56 participants with SZ, 33 with bipolar I disorder, and 58 HC who completed the Ekman task; with a sub-set of 47 SZ, 27 BD, and 47 HC completing the TASIT. Of these, 32 SZ, 24 BD, and 36 HC completed the CERQ. All subjects fulfilled relevant DSM-IV criteria. Healthy participants had no personal history of a DSM-IV Axis 1 disorder other than anxiety disorders, and no history of psychosis (SZ or BD) in their first-degree biological relatives. Exclusion criteria included inability to communicate sufficiently in English, current neurological disorder and/or having been treated with electro convulsive therapy (ECT) in the previous 6 months.

Participants were recruited from a number of sources, including the Australian Schizophrenia Research Bank (ASRB; Loughland et al., 2011), the Sydney Bipolar Disorder Clinic (Mitchell et al., 2009), and advertisements in the local community and newspaper. The SZ group consisted of 32 males (57.1%) and 24 females (42.9%), aged 19–63 years (*M* = 44.57, SD = 10.37). The BD group comprised 18 males (54.3%) and 15 females (45.7%), aged 22–60 years (*M* = 40.67, SD = 11.27). At the time of assessment, 12 BD participants were determined euthymic (36.4%), 12 hypomanic (36.4%), 1 depressed (3%), and 8 in a mixed state (24.2%). The HC participants were equally divided between males and females and aged 19–61 years (*M* = 33.91, SD = 12.24). There were missing data on less than 10% of items for seven clinical participants; missing data were replaced with the group median for each item.

A number of SZ and BD participants were prescribed antipsychotic, antidepressant, or mood stabilizing medication at the time of testing. Of the SZ patients, 18 were taking an antipsychotic, 18 an antipsychotic and antidepressant, 5 an antipsychotic and mood stabilizer, and 7 an antipsychotic and antidepressant plus a mood stabilizer. Of the BD participants, 2 were taking an antipsychotic, 7 a mood stabilizer, 10 an antipsychotic and a mood stabilizer, 7 an antidepressant and mood stabilizer, and 2 were taking all three. The remaining patients were not currently taking any medication.

### Materials

#### Internal state scale

Mood state of the BD participants at the time of assessment was indexed by the Internal State Scale (ISS; Bauer et al., 1991) The ISS is a 16-item self-report scale that provides an index of manic and depressive symptomatology for the preceding 24 h. The measure is divided into four subscales: *Activation*, *Perceived Conflict*, *Well-Being*, and a *Depression Index*. Each item is rated on a 100-point visual analog scale anchored at 0 and 100, with each anchor incorporating both the frequency and severity of the symptom. The Activation subscale items correspond to manic symptoms (e.g., “My thoughts are going fast), and the Depression Index to depressive symptoms (e.g., “It seems like nothing will work out for me”), and they have been found to correlate highly with clinician ratings of mania and depression, respectively.

#### Positive and Negative Syndrome Scale

Current symptoms of SZ and BD patients were assessed using the Positive and Negative Syndrome Scale (PANSS; Kay et al., 1987). The PANSS has three subscales, *positive symptoms*, *negative symptoms*, and *general psychopathology*. Each symptom receives a rating between 1 (*Absent*) and 5 (*Severe*).

#### Ekman 60-faces task from the Facial Expressions of Emotion: Stimuli and Tests

As a measure of emotion perception, subjects were administered the Ekman 60-faces task from the FEEST (Young et al., 2002). FEEST is a computerized task, where participants are required to identify six basic emotions from the Ekman and Friesen (1976) series (happiness, sadness, anger, fear, surprise, and disgust) presented in a still “photographic” format. In accordance with previous research of emotion recognition, happiness and surprise were considered “positive” expressions, and sadness, anger, fear, and disgust were considered “negative” expressions.

#### The Awareness of Social Inference Test

All subjects completed Form A of TASIT (McDonald et al., 2003) to index emotion perception and ToM abilities. TASIT comprises three parts, as detailed below, and an administration time of approximately 35 min. Practice items were provided for all parts, and the videotape was paused after each video clip to allow participants time to comprehend and answer the questions.

Part 1: *The Emotion Evaluation Test* comprises 28 short video clips in which an actor portrays one of six basic emotions (happy, sad, fear, disgust, surprise, or anger). The maximum attainable accuracy score is 28.

Part 2: *Social Inference – Minimal* is comprised of 15 video clips depicting sincere and sarcastic (simple sarcasm and paradoxical sarcasm) interaction between two actors, thus examining ToM. The dialog used is ambiguous, requiring attendance to general demeanor, tone of voice, facial expressions, and/or gestures, in order to interpret the situation. In sincere exchanges, the target actors mean what they say. In simple sarcasm exchanges, one of the target actors means the opposite of what is said, and intends for the listener to comprehend the real meaning of what is said. In paradoxical sarcasm exchanges, the dialog between speakers is nonsensical unless it is understood that one speaker is being sarcastic. At the end of each clip, participants answered four questions designed to elicit interpretations of what the speaker was thinking, doing (e.g., criticizing), meaning to say, and feeling. As each of the 15 video clips is given a score out of 4, the maximum score attainable in this part is 60.

Part 3: *Social Inference – Enriched*, which also examines ToM, is comprised of 16 vignettes for which participants are provided extra information about the true state of affairs before or after the dialog of interest. Participants answer four questions designed to examine their ability to detect deception in social encounters (i.e., lies) and sarcasm. The maximum attainable score in this section is 64.

#### Cognitive Emotion Regulation Questionnaire

The Cognitive Emotion Regulation Questionnaire (CERQ) measures various types of cognitive strategies employed to regulate emotion in response to the experience of threatening or stressful life events (Garnefski et al., 2001). The CERQ is a 36-item questionnaire, consisting of nine conceptually distinct subscales (four items each), each pertaining to a particular type of regulatory strategy. A person’s tendency to engage in each strategy is measured on a five-point Likert scale ranging from 1 (almost never) to 5 (almost always). Individual subscale scores are obtained by summing the scores for each strategy (ranging from 4 to 20); the higher the subscale score, the more often the cognitive strategy is used. The four maladaptive subscales of the CERQ include: *self-blame* (thoughts of blaming yourself for what you have experienced), *other-blame* (thoughts of blaming another person for what you have experienced), *rumination* (thinking about feelings and thoughts associated with the negative event), and *catastrophizing* (thoughts that over-emphasize the significance and extent of the experience). The five positive subscales include: *putting into perspective* (thoughts that minimize the seriousness of the event relative to other life events), *positive refocusing* (distracting oneself from thinking about the event by focusing on positive thoughts or issues), *positive reappraisal* (reframing the event in a positive light), *acceptance* (accepting the experience and resigning oneself to what has happened), and *refocus on planning* (thinking about how to handle the negative event and what steps to take). Internal consistencies of CERQ subscales range from 0.68 to 0.83 (Garnefski et al., 2001), and evidence for discriminant and convergent validity has been reported (Garnefski et al., 2004, 2005).

### Procedure

Subjects were tested individually in a dedicated testing laboratory. Administration time was approximately 3 h. Participants were reimbursed for their time and travel expenses.

## Results

Descriptive statistics for demographic and clinical data collected using the PANSS are presented in Table 1. One-way analysis of variance (ANOVA) was conducted to investigate group differences in age and a Chi-squared analysis to examine gender distribution. There were no differences in sex distribution among the three groups. However, there was a significant group difference in age (see Table 1); Tukey’s HSD tests revealed that both SZ (*p* < 0.0001) and BD (*p* < 0.01) groups were significantly older than the HC group, but the SZ and BD groups did not significantly differ in age (*p* = 0.262). Age was thus employed as a covariate in focal analyses of the clinical and HC groups. Unsurprisingly, a significant difference in PANSS scores was found between the SZ and BD groups, where SZ participants reported significantly higher levels of symptoms than the BD participants on all three subscales; positive symptoms (*F*_1,88_ = 27.54, *p* < 0.0001; η^2^ = 0.240), negative symptoms (*F*_1,88_ = 20.57, *p* < 0.0001; η^2^ = 0.191), and general symptoms (*F*_1,88_ = 7.76, *p* = 0.007; η^2^ = 0.082).

**Table 1:** **Demographic information and PANSS scores for each group**.

	SZ (*n* = 56)	BD-I (*n* = 33)	HC (*n* = 58)	Statistical values for main effects	Effect size	Direction of significant group differences
	
Age (years)	44.57 (10.37)	40.67 (11.27)	33.91 (12.24)	*F*_2,146_ = 12.81, *p* < 0.0001	η^2^ = 0.151	SZ > HC***, BD > HC*
Gender (M/F)	32/24	18/15	29/29	χ^2^ = 0.596, *p* = 0.742		ns
PANSS positive symptoms	16.32 (7.09)	9.36 (3.58)		*F*_1,88_ = 27.54, *p* < 0.0001	η^2^ = 0.240	SZ > BD***
PANSS negative symptoms	17.34 (7.50)	10.91 (4.09)		*F*_1,88_ = 20.57, *p* < 0.0001	η^2^ = 0.191	SZ > BD***
PANSS general symptoms	31.14 (9.84)	25.88 (5.93)		*F*_1,88_ = 7.76, *p* = 0.007	η^2^ = 0.082	SZ > BD**
PANSS total	64.80 (19.68)	46.64 (10.55)		*F*_1,88_ = 20.57, *p* < 0.0001	η^2^ = 0.216	SZ > BD***

***p < 0.001, **p < 0.01, and *p < 0.05.

*PANSS, Positive and Negative Syndrome Scale*.

### Group differences in performance on the Ekman 60-faces task

To investigate group differences in Ekman performance, we conducted a multivariate analysis of variance (MANOVA) with group (SZ, BD, and HC) as the independent variable and accuracy on the Ekman 60-faces task entered as dependent variables. Group means and SD for facial emotion recognition accuracy are presented in Table 2. There was a significant main effect of group for the recognition accuracy of positive, negative, and total emotions (see Table 2). Subsequent univariate analyses of covariance (ANCOVAs) were used to examine pairwise group differences between individual patient groups and controls, controlling for age. These analyses revealed that, in comparison to the HC group, SZ participants demonstrated impairments in the recognition of positive (*F*_2,113_ = 4.44, *p* = 0.037; partial η^2^ = 0.038) and negative emotions (*F*_2,113_ = 8.45, *p* = 0.004; partial η^2^ = 0.071), as well as overall emotion recognition (*F*_2,113_ = 9.23, *p* = 0.003; partial η^2^ = 0.077). ANOVAs examining clinical group differences showed poorer performance in the SZ than the BD group in negative (*F*_1,88_ = 9.10, *p* = 0.004; η^2^ = 0.094) and overall emotion recognition (*F*_1,88_ = 8.47, *p* = 0.005; η^2^ = 0.089). There were no significant differences in emotion recognition accuracy between the BD and HC groups.

**Table 2:** **Means and SD for all groups on the social cognition measures**.

	SZ	BD	HC	Statistical values for main effects	Effect size	Direction of significant group differences
**Ekman subscale and total scores**	**(*n* = 56)**	**(*n* = 33)**	**(*n* = 58)**

Positive emotions	17.89 (2.35)	18.61 (1.48)	18.84 (1.58)	*F*_2,146_ = 3.80, *p* = 0.025	η^2^ = 0.050	SZ < HC*
Negative emotions	26.95 (6.92)	30.88 (3.85)	30.38 (4.44)	*F*_2,146_ = 7.80, *p* = 0.001	η^2^ = 0.098	SZ < BD**; SZ < HC**
Overall total	44.84 (8.63)	49.48 (3.99)	49.22 (4.71)	*F*_2,146_ = 8.55, *p* < 0.0001	η^2^ = 0.106	SZ < BD**; SZ < HC**

**TASIT subscale and total scores**	**(*n* = 47)**	**(*n* = 27)**	**(*n* = 47)**	

Part 1	22.11 (4.17)	23.52 (2.82)	24.62 (2.72)	*F*_2,120_ = 6.524, *p* = 0.002	η^2^ = 0.101	SZ < HC***
Part 2	45.68 (9.35)	53.37 (4.99)	53.68 (5.97)	*F*_2,120_ = 16.75, *p* < 0.0001	η^2^ = 0.221	SZ < BD***; SZ < HC***
Part 3	48.19 (6.69)	52.33 (6.78)	55.96 (5.54)	*F*_2,120_ = 17.93, *p* < 0.0001	η^2^ = 0.233	SZ < BD*; SZ < HC***; BD < HC*
Overall total	115.98 (16.97)	129.22 (12.28)	134.26 (12.01)	*F*_2,120_ = 20.34, *p* < 0.0001	η^2^ = 0.256	SZ < BD***; SZ < HC***

***p < 0.001, **p < 0.01, and *p < 0.05.

*Ekman, Ekman 60-faces task; TASIT, The Awareness of Social Inference Test*.

### Group differences in TASIT performance

Group means and SD for TASIT performance are summarized in Table 2. To investigate group differences in TASIT performance, we conducted a MANOVA with group as the independent variable (SZ, BD, and HC), and TASIT subscale (Part 1 – Emotion Evaluation Test, Part 2 – Social Inference: Minimal, and Part 3 – Social Inference: Enriched) and overall total scores entered as dependent variables. There was a significant main effect of group for all four scores; Part 1, Part 2, Part 3, and overall total score (see Table 2). Subsequent ANCOVAs examining specific group differences between the clinical and control groups, controlling for age, showed that in comparison to the HC group SZ participants demonstrated impairments in every component of the TASIT: Part 1 (*F*_2,93_ = 4.52, *p* = 0.036; partial η^2^ = 0.047); Part 2 (*F*_2,93_ = 18.54, *p* < 0.0001; partial η^2^ = 0.169); and Part 3 (*F*_2,93_ = 22.03, *p* < 0.0001; partial η^2^ = 0.195). The only difference between scores for the BD and HC groups was on Part 3, with BD participants showing deficits on the threshold of significant (*F*_2,73_ = 3.97, *p* = 0.05; partial η^2^ = 0.053). Further ANOVAs undertaken for pairwise comparisons of the clinical groups showed that SZ had more difficulty performing than BD patients on Part 2 (*F*_1,73_ = 15.65, *p* < 0.0001; η^2^ = 0.179), Part 3 (*F*_1,73_ = 6.51, *p* = 0.013; η^2^ = 0.083), and overall (*F*_1,73_ = 12.62, *p* = 0.001; η^2^ = 0.149).

### Frequency of use of Cognitive Emotion Regulation Questionnaire strategies

Group means and SD for CERQ subscales are reported in Table 3. To investigate group differences in the frequency of use of CERQ strategies (represented by total scores on each subscale), we conducted a MANOVA with group (SZ, BD, and HC) as the independent variable, and total scores on each of the nine CERQ subscales entered as dependent variables. This revealed a significant main effect of group for rumination, positive reappraisal, blaming others, catastrophizing, and self-blame, but not for acceptance, putting into perspective, positive refocusing, or refocus on planning (see Table 3).

**Table 3:** **Means and SD for all groups on the CERQ**.

CERQ subscale	SZ (*n* = 32)	BD (*n* = 24)	HC (*n* = 36)	Statistical values for main effects	Effect size	Direction of significant group differences
Rumination	14.56 (5.95)	13.54 (3.31)	11.69 (3.65)	*F*_2,91_ = 3.53, *p* = 0.034	η^2^ = 0.073	SZ > HC**; BD > HC**
Positive reappraisal	14.13 (4.11)	12.58 (4.09)	15.50 (3.12)	*F*_2,91_ = 4.40, *p* = 0.015	η^2^ = 0.091	BD < HC*
Other-blame	10.59 (4.53)	8.04 (2.85)	8.61 (3.21)	*F*_2,91_ = 4.01, *p* = 0.021	η^2^ = 0.083	SZ > BD**; SZ > HC**
Acceptance	13.66 (3.41)	12.71 (3.21)	12.86 (3.28)	*F*_2,91_ = 0.71, *p* = 0.492		ns
Catastrophizing	11.31 (4.37)	10.33 (4.29)	8.25 (3.24)	*F*_2,91_ = 5.36, *p* = 0.006	η^2^ = 0.107	SZ > HC**; BD > HC**
Putting into perspective	13.06 (3.89)	13.71 (8.67)	15.14 (3.62)	*F*_2,91_ = 1.28, *p* = 0.282		ns
Positive refocusing	11.66 (3.69)	10.00 (4.17)	12.00 (3.38)	*F*_2,91_ = 2.25, *p* = 0.111		ns
Refocus on planning	15.59 (7.64)	13.42 (3.83)	15.44 (2.87)	*F*_2,91_ = 1.43, *p* = 0.244		ns
Self-blame	11.94 (3.41)	12.75 (4.95)	10.33 (3.24)	*F*_2,91_ = 3.19, *p* = 0.046	η^2^ = 0.067	BD > SZ* > HC**

**p < 0.01 and *p < 0.05.

*CERQ, Cognitive Emotion Regulation Questionnaire*.

In focal analyses of clinical and control groups on the significant CERQ subscales, a series of ANCOVAs were undertaken to examine pairwise group differences in the frequency of use of these strategies, controlling for age. In comparison to the HC group, SZ participants were more likely to engage in *catastrophizing* (*F*_2,67_ = 10.78, *p* = 0.002; partial η^2^ = 0.142) and *rumination* (*F*_2,67_ = 3.99, *p* = 0.05; partial η^2^ = 0.058), while BD participants reported less use of *positive reappraisal* (*F*_2,59_ = 9.38, *p* = 0.003; partial η^2^ = 0.141) and greater use of *self-blame* (*F*_2,59_ = 5.15, *p* = 0.027; partial η^2^ = 0.083). An ANOVA comparing the patient groups showed SZ participants were more likely to employ the regulatory strategy of *blaming others* than BD participants (*F*_2155_ = 5.86, *p* = 0.019; η^2^ = 0.098).

### Association of social cognitive performance with CERQ

Zero-order correlations between social cognitive performance and CERQ subscales were carried out using Pearson’s product-moment correlations. Associations between emotion recognition accuracy on the Ekman task and CERQ subscales are reported in Table 4. The only significant correlations were for the HC group, with the Ekman positive emotions subscale score showing positive associations with the CERQ subscales *acceptance* (*r* = 0.501, *p* = 0.002) and *putting into perspective* (*r* = 0.429, *p* = 0.009). There were no significant correlations for either the SZ or BD groups. Subsequently, statistical comparison of correlation coefficients for independent samples was conducted via the Fisher *z transformation of r* procedure (Cohen and Cohen, 1983) to investigate pairwise group differences in the Ekman scores and CERQ subscales that were correlated for controls but not for patients. For all comparisons between the SZ and HC groups there was a significant difference between correlations (positive emotions with: acceptance *Z* = −2.45, *p* = 0.014; putting into perspective *Z* = −2.14, *p* = 0.032). For BD and HC, the correlations were also found to differ significantly between groups (positive emotions with: acceptance *Z* = −2.40, *p* = 0.016; putting into perspective *Z* = −2.21, *p* = 0.027). There was no significant difference between SZ and BD in the correlations for these variables.

**Table 4:** **Pearson’s two-tailed correlations between Ekman scores and CERQ strategies for SZ, BD, and HC groups**.

CERQ subscales	Ekman 60-faces scores								
	Positive emotions			Negative emotions			Total score		
	SZ	BD	HC	SZ	BD	HC	SZ	BD	HC
Rumination	−0.180	0.073	0.124	−0.096	−0.254	0.137	−0.129	−0.214	0.165
Positive Reappraisal	−0.123	−0.084	−0.003	−0.135	−0.027	0.050	−0.148	−0.053	0.045
Other-blame	−0.265	0.227	0.134	−0.163	−0.382	−0.215	−0.209	−0.284	−0.156
Acceptance	−0.073	−0.120	0.501**	−0.159	−0.118	0.077	−0.155	−0.151	0.229
Catastrophizing	−0.245	0.080	0.052	−0.279	−0.113	−0.282	−0.303	−0.080	−0.243
Putting in perspective	−0.086	−0.156	0.429**	−0.192	0.066	0.266	−0.188	0.011	0.380
Positive refocusing	−0.098	−0.202	−0.134	0.010	−0.190	0.014	−0.016	−0.246	−0.029
Refocus on planning	−0.273	−0.083	0.183	−0.102	−0.115	0.035	−0.158	−0.136	0.090
Self-blame	0.055	0.084	0.273	−0.153	−0.108	−0.106	−0.117	−0.074	−0.012

**p < 0.01.

*CERQ, Cognitive Emotion Regulation Questionnaire*.

Similarly, significant correlations between TASIT scores and CERQ subscales were found for the HC group only, as reported in Table 5. Significant negative associations were found in the HC group between TASIT Part 2 scores and the frequent use of *rumination* (*r* = −0.452, *p* = 0.008), *catastrophizing* (*r* = −0.476, *p* = 0.005), and *blaming others* (*r* = −0.498, *p* = 0.003). TASIT Part 3 showed negative correlations with *catastrophizing* (*r* = −0.538, *p* = 0.001) and *blaming others* (*r* = −0.451, *p* = 0.008) as did overall TASIT performance with *catastrophizing* (*r* = −0.549, *p* = 0.001) and *blaming others* (*r* = −0.506, *p* = 0.003). Again, there were no significant correlations for either the SZ or BD groups. Statistical comparison of correlation coefficients for independent samples was subsequently conducted to investigate pairwise group differences in these associations. For TASIT Part 2, the correlations with both rumination (*Z* = 3.06, *p* = 0.002) and catastrophizing (*Z* = 4.48, *p* < 0.0001) differed significantly between the BD and HC groups, as did the correlations of TASIT Part 3 with catastrophizing (*Z* = 2.92, *p* = 0.004). The associations between TASIT scores and blaming others showed no significant group differences in coefficients. None of the comparisons of correlations between SZ and HC or SZ and BD groups were significant.

**Table 5:** **Pearson’s two-tailed correlations between TASIT scores and CERQ strategies for SZ, BD, and HC groups**.

CERQ subscales	TASIT subscales														
	Part 1			Part 2			Part 3			Total		
	SZ	BD	HC	SZ	BD	HC	SZ	BD	HC	SZ	BD	HC
Rumination	−0.044	0.231	−0.184	−0.192	0.376	−0.452**	−0.376	0.305	−0.293	−0.272	0.369	−0.396
Positive reappraisal	−0.063	−0.085	0.015	−0.344	0.007	0.087	−0.258	−0.074	−0.015	−0.310	−0.055	0.038
Other-blame	−0.261	−0.065	−0.247	−0.091	−0.086	−0.498**	−0.204	−0.164	−0.451**	−0.177	−0.138	−0.506**
Acceptance	−0.252	0.203	0.042	−0.233	−0.098	−0.007	−0.276	−0.05	−0.040	−0.284	−0.028	−0.013
Catastrophizing	−0.222	0.135	−0.299	−0.092	0.065	−0.476**	−0.268	0.238	−0.538***	−0.198	0.185	−0.549***
Putting in perspective	−0.052	−0.013	0.018	−0.263	0.100	0.130	−0.151	0.018	0.154	−0.217	0.048	0.139
Positive refocusing	0.131	−0.185	0.090	0.054	−0.167	−0.002	0.087	−0.290	−0.124	0.087	−0.265	−0.038
Refocus on planning	−0.286	−0.102	−0.014	−0.044	0.079	0.112	0.064	−0.120	−0.057	−0.040	−0.054	0.024
Self-blame	−0.083	0.336	−0.177	−0.069	0.191	−0.285	−0.091	0.294	−0.224	−0.089	0.309	−0.282

***p < 0.001 and **p < 0.01.

*TASIT, The Awareness of Social Inference Test; CERQ, Cognitive Emotion Regulation Questionnaire*.

### Social cognitive predictors of frequency of use of CERQ strategies

To further examine the pattern of correlations between social cognitive performance and the CERQ, a series of nine stepwise multiple regression analyses were conducted to test which of the social cognition scores best predicted frequency of use of CERQ strategies in the three experimental groups, using the CERQ subscales as dependent variables, controlling for age. Performance scores on each of the Ekman and TASIT subscales were entered as independent variables; significance level for entry was set at 0.01 to reduce type-I error. As the correlations also show the existence of collinearity among social cognition subscales, diagnostics for collinearity of Ekman, and TASIT predictors were therefore considered in subsequent regression analyses by Variance Inflation Factor (VIF) scores, where VIF scores above 10 are considered of serious concern. No VIF score was found to be above 3.

In support of the correlational pattern, these analyses yielded significant regression models for the HC group only, with no variables reaching significance for entry into any model for either the SZ or BD groups. For the HC group, positive emotion recognition accuracy on the Ekman task was found to explain 20.8% of the variance in the frequency of use of *acceptance* (*F*_1,32_ = 9.43, *p* = 0.004; Adjusted *R*^2^ = 0.208). Performance on TASIT Part 2 was found to explain 17.9% of the variance in the frequency of use of *rumination* (*F*_1,32_ = 7.98, *p* = 0.008; Adjusted *R*^2^ = 0.179) and 22.3% of the variance in the use of *blaming others* (*F*_1,32_ = 10.20, *p* = 0.003; Adjusted *R*^2^ = 0.223). TASIT Part 3 scores explained 26.6% of the variance in frequency of use of *catastrophizing* (*F*_1,32_ = 7.69, *p* = 0.001; Adjusted *R*^2^ = 0.266).

## Discussion

This study set out to examine the relationship between social cognitive abilities and the frequency of use of cognitive strategies for regulating negative emotion in patients with SZ and BD. We firstly examined performance on social cognitive tasks of emotion perception and higher order social cognition (i.e., ToM) between the clinical and control groups. As predicted, SZ participants’ demonstrated greater impairments in social cognition (encompassing both basic emotion processing and ToM) relative to BD, who only showed impairment on higher order social cognition on the TASIT (but not basic emotion perception) compared to HC. In addition, there were distinct patterns of CERQ performance in both patient groups, with both SZ and BD groups reporting more frequent use of maladaptive emotion regulation strategies (namely, rumination, catastrophizing, and self-blame), as well as less use of the adaptive strategy of putting into perspective, relative to HC. Examination of the relationship between social cognition and cognitive emotion regulation revealed associations for HC participants only, with higher levels of emotion recognition being associated with greater use of the strategy of putting into perspective, and better TASIT performance associated with less frequent use of rumination, catastrophizing, and blaming others. Contrary to predictions, no associations between social cognitive performance and cognitive regulatory strategies were revealed for either the SZ or BD groups. Further investigation of the differences in correlation coefficients for those performance scores and CERQ subscales that were correlated for controls but not for patients showed the relationships between positive emotion recognition and the use of adaptive reframing strategies differed between the clinical and HC groups, while the relationships between ToM abilities and the use of maladaptive strategies (rumination and catastrophizing) differed between the BD and HC groups. Additionally, there was no difference between the clinical groups in the correlations for these variables. Finally, with regard to the predictive utility of social cognitive performance: within the control group, recognition accuracy of positive emotions predicted greater use of acceptance, with the addition of poor TASIT performance predicting increased use of rumination, blaming others, and catastrophizing.

The present findings for social cognitive impairments in SZ and BD converge with previous evidence for emotion perception deficits in SZ (Addington and Addington, 1998; Edwards et al., 2002; Sparks et al., 2010), as well as a number of studies showing ToM deficits in SZ and BD (Addington and Addington, 1998; Kerr et al., 2003; Bora et al., 2005; Brune, 2005; Harrington et al., 2005; Olley et al., 2005; Montag et al., 2010). Our results for SZ are especially consistent with recent studies showing impaired sarcasm perception (Kern et al., 2009; Sparks et al., 2010) and social context processing (Green et al., 2007b, 2008). However, in contrast to previous studies where both SZ and BD patients have shown impaired facial emotion perception in comparison to community controls (Addington and Addington, 1998), there were no such deficits in the BD group. Additionally, the SZ group was found to be impaired on both the Ekman and TASIT tasks when compared to the BD group. It thus appears that the basic emotion perception and ToM abilities of SZ patients are compromised not only in relation to non-patient controls but also in comparison to patients with BD.

With regard to the frequency of use of particular cognitive strategies employed to regulate negative emotion, the tendency for SZ and BD patients to employ maladaptive approaches to regulate affect is consistent with the style demonstrated in other studies (Green et al., 2011; Rowland et al., in revision), and in other disorders such as depression (Garnefski et al., 2002). One distinction is that SZ participants employed other-blame more frequently than both BD and HC groups, while BD participants uniquely demonstrated greater use of self-blame compared to both SZ and HC groups, less positive refocusing compared to the SZ group, and less positive reappraisal than controls. These findings are also consistent with previous evidence (Livingstone et al., 2009; Rowland et al., in revision).

In contrast to our predictions, examination of the relationship between social cognition and emotion regulation revealed associations for HC participants only, with no associations present in either the SZ or BD groups. However, the specific associations found for the HC group were as expected, with the recognition of positive emotions found to be positively associated with the CERQ strategy of acceptance, and (both positive and overall) emotion recognition showing positive associations with the tendency to put things into perspective. Furthermore, within the control group, recognition accuracy of positive emotions actually predicted greater use of the positive reframing strategy of acceptance. This suggests that for people with no history of mental illness, the ability to accurately recognize facial affect expression, and positive affect in particular, is related to more frequent use of adaptive cognitive reframing strategies. In addition, for HC there was also a negative association between TASIT performance and frequent use of rumination, catastrophizing, and blaming others. Of these, performance on Part 2 of the TASIT (*Social Inference – Minimal*) was most predictive of the use of rumination and blaming others, while Part 3 (*Social Inference – Enriched*) performance best predicted use of catastrophizing, indicating that accurate social cognitive skills are associated with less frequent use of these maladaptive emotion regulation strategies. While these findings confirm expected relationships between social cognitive skills and the capacity to implement adaptive strategies for emotion regulation in non-clinical (healthy) individuals, they also highlight the lack of “normal” associations between social cognition and emotion regulatory styles in SZ and BD. In the context of evidence that a number of these associations differ significantly between the clinical and control groups, it appears that known links between social cognition and the capacity to self-regulate negative emotion are not intact in psychotic individuals.

The present findings may be limited by a number of factors. First, it is possible that the limited range of scores for SZ or BD on some social cognitive measures could have impeded the capacity to reveal significant associations between these measures and cognitive regulatory strategies. This is plausible in the context of the significant associations found for HC. This may also be related to the relatively small sample sizes for each participant group, which may have limited the power to detect within-group associations between social cognition and CERQ strategies, and which precluded analyses of the effects of illness phases (e.g., depressed, euthymic, and manic) of the BD group in particular. Secondly, it should be noted that TASIT performance relies heavily on the interpretation of sarcasm in scenarios involving fluent English speakers, and we do not know whether these findings involving TASIT would translate across cultures. Thirdly, the reliance on self-report to measure self-regulation of affect can be problematic in that individuals may not always be consciously aware or sure of their own use of cognitive emotion regulation strategies in stressful situations; these may be context-dependent or unconsciously triggered and can be impacted by memory biases. Additionally, the CERQ only assesses regulation of negative emotion, and does not take into account strategies utilized in response to positive events. A potentially serious limitation of this study is that we have not examined the effects of medication on our results; given that many SZ and BD patients were receiving pharmacotherapy, future studies would benefit from further investigation of these factors. Fourth, since the participants in this study were outpatients living in the community, it may not be appropriate to generalize the implications of the present findings to individuals who are experiencing acute symptoms or those residing in inpatient settings. Finally, we note that the large number of analyses performed in this study precluded the utility of a formal correction for multiple testing; despite setting a reduced significance level (*p* < 0.01), it is possible that some spurious associations were revealed in this study. Further research with larger samples, testing strict hypotheses (rather than exploratory analyses as presented here), to replicate current findings, will be necessary to determine the reliability of the current results.

In summary, the present findings are consistent with previous evidence for social cognitive impairments and maladaptive patterns of emotion regulation in SZ and BD. We aimed to examine associations between social cognitive performance and emotion regulatory styles in SZ and BD, however the expected associations were revealed for the HC group only. It is possible that competency in the execution of specific emotion regulation strategies may rest on other neuropsychological skills (e.g., executive functioning) not tested here, rather than being mediated via social cognitive skills in BD and SZ. The lack of association between social cognition and cognitive emotion regulation in the patient groups is, however, consistent with a lack of integrity of fronto-limbic brain networks (Morris et al., 2012). The current findings suggest that dysfunction in these brain networks may underpin both social cognitive deficits and regulatory capacities, albeit via different routes. Further investigation of the association between social cognitive skills and emotion regulation capacities in psychotic disorders, ideally integrating neurofunctional and neuroanatomical data, is warranted.

## Conflict of Interest Statement

The authors declare that the research was conducted in the absence of any commercial or financial relationships that could be construed as a potential conflict of interest.
